# Semi-supervised tissue segmentation from histopathological images with consistency regularization and uncertainty estimation

**DOI:** 10.1038/s41598-025-90221-x

**Published:** 2025-02-22

**Authors:** G. V. S. Sudhamsh, S. Girisha, R. Rashmi

**Affiliations:** 1 Department of Computer Science and Engineering, Manipal Institute of Technology Bengaluru, Manipal Academy of Higher Education, Manipal, India; 2 Department of Data Science and Computer Applications, Manipal Institute of Technology, Manipal Academy of Higher Education, Manipal, India

**Keywords:** Computer Aided Diagnostic Systems, Deep Learning, Histopathological Image Analysis, Semantic Segmentation, Semi-Supervised Learning, Image processing, Biomedical engineering

## Abstract

Pathologists have depended on their visual experience to assess tissue structures in smear images, which was time-consuming, error-prone, and inconsistent. Deep learning, particularly Convolutional Neural Networks (CNNs), offers the ability to automate this procedure by recognizing patterns in tissue images. However, training these models necessitates huge amounts of labeled data, which can be difficult to come by due to the skill required for annotation and the unavailability of data, particularly for rare diseases. This work introduces a new semi-supervised method for tissue structure semantic segmentation in histopathological images. The study presents a CNN based teacher model that generates pseudo-labels to train a student model, aiming to overcome the drawbacks of conventional supervised learning approaches. Self-supervised training is used to improve the teacher model’s performance on smaller datasets. Consistency regularization is integrated to efficiently train the student model on labeled data. Further, the study uses Monte Carlo dropout to estimate the uncertainty of proposed model. The proposed model demonstrated promising results by achieving an mIoU score of 0.64 on a public dataset, highlighting its potential to improve segmentation accuracy in histopathological image analysis.

## Introduction

Analyzing tissue structures in pathology traditionally involved labor-intensive and subjective methods. Pathologists manually identified and outlined specific structures in histopathological images, relying heavily on their expertise and visual inspection^1,2^. This manual technique is acknowledged for its tendency to be time-consuming, vulnerable to human error, and lacks consistency across different experts^3^. Additionally, the reliance on predefined features limits the adaptability of these methods to diverse tissue appearances and complex structural variations commonly found in medical imaging.

The advancement of deep learning, especially CNNs, has led to a notable revolution in the segmentation of histopathological images^4,5^. Unlike traditional methods, CNNs learn intricate patterns and variations directly from data through convolution, capturing subtle nuances in tissue morphology, such as color variations, texture differences, and spatial relationships^6^. Consequently, CNN-based methods achieve superior segmentation accuracy and consistency. However, training CNNs effectively requires large amounts of labeled data, where each pixel in histopathological images is meticulously annotated with its corresponding class label. Acquiring such annotated datasets is challenging in the medical domain, as the annotation process is expensive and demands the expertise of specialized pathologists^6^. Data scarcity is also a significant concern, particularly for rare diseases or specialized tissue types^7,8^.

The rationale behind this research originates from the crucial necessity to enhance the accuracy and effectiveness of histopathological image analysis. Effective tissue segmentation is essential for various diagnostic and research applications, including identifying cancerous regions, quantifying disease progression, and understanding tissue architecture. Traditional methods fall short in providing the necessary precision and adaptability, highlighting the importance of developing advanced techniques^9^. The limitations of traditional methods, such as their dependency on manual intervention, susceptibility to human error, and inability to handle complex and varied tissue structures, underscore the need for innovative solutions^10^. By leveraging CNNs and addressing the challenges of data scarcity and annotation, this study aims to enhance the segmentation process, ultimately contributing to more reliable and efficient pathology practices^11^.

The limited availability of labeled data has driven researchers to explore semi-supervised learning as an alternative approach^12^. Approaches based on utilizing both labeled and unlabeled data samples, semi-supervised learning leverages their combined strengths, enhancing the learning process^13^. Labeled data provides crucial guidance through explicit annotations, while abundant and easily accessible unlabeled data contributes significant additional information. By strategically utilizing unlabeled data, semi-supervised learning reduces the dependence on fully annotated datasets, making the training process more efficient and cost-effective.

This research focuses on developing a semi-supervised semantic segmentation model for tissue delineation from histopathological images. A lightweight CNN model is presented as the teacher model for generating pseudo-labels, which are subsequently used to train the student model. The lightweight characteristics of the teacher model facilitate training on smaller datasets. Additionally, we utilize a self-supervised training approach to efficiently train the teacher model. The student model is designed to capture both spatial and contextual information efficiently. Our proposed semi-supervised framework effectively trains the student model, and we evaluate the model on a public dataset, demonstrating promising results.

This paper’s contributions include the following:

- The study introduces a teacher model optimized for generating pseudo-labels from unlabeled training dataset, enhancing the efficiency of the labeling process.
- The study utilizes Monte Carlo dropout method to evaluate the uncertain nature of teacher model in generating pseudo-labels, ensuring the generation of reliable pseudo-labels.
- The study introduces a student model designed to efficiently capture and extract both spatial and contextual features from input histopathological images, resulting in precise segmentation maps.
- The study proposed an innovative semi-supervised learning framework that leverages consistency regularization to effectively train the student model, improving segmentation accuracy with limited labeled data.

The remainder of this paper is organized as follows: sections “Related works”, “Methodology” and “Results and discussion” presents the related works, proposed methodology and results obtained on public dataset respectively. Finally, the conclusion of the study are provided in section “Conclusion”.

## Related works

Segmenting tissues from histopathological images is an essential aspect of medical image analysis. Researchers have explored two main approaches: classical methods and deep learning techniques. This section summarizes approaches used for tissue segmentation from histopathological images. Classical methods for tissue segmentation typically rely on a series of stages, extensively using handcrafted features. These methods often start with preprocessing steps to enhance image quality, followed by feature extraction and classification or clustering to identify tissue structures^14^. Despite their widespread use, classical image processing techniques face several limitations that hinder their effectiveness and generalizability to new datasets^15^. In contrast, deep learning based methods automatically learn intricate patterns and variations directly from the data, addressing the limitations of classical methods and providing a more effective solution for tissue segmentation tasks^16^.

### Deep learning techniques

Deep learning methods, particularly CNNs have revolutionized histopathological image analysis by their capability to learn high-level features directly from images on their own. Unlike classical methods that rely on handcrafted features and manual tuning, CNNs allow for the capture of complex patterns and subtle variations in tissue morphology^9,11^. Architectures like VGG16^17^, ResNets^18^, and Inception^19^ have shown remarkable performance in image classification and segmentation tasks, leveraging their deep layers to learn rich and hierarchical features. U-Net^20^, a widely used model for the segmentation of different structures in medical images, designed to grasp the full spectrum of features, from minute details to broader structures. However, U-Net and similar architectures^21^ often face semantic gap issues between the encoder and decoder, where the detailed spatial information may be lost during the down-sampling process^22^.

To mitigate these challenges, researchers have developed various enhancements to the U-Net architecture^23–25^. Incorporating atrous spatial pyramid pooling layers helps capture multi-scale contextual information, while attention modules focus on relevant parts of the image, enhancing feature representation^26–28^. Transformers have demonstrated effectiveness in natural language processing, are also being integrated into CNNs to enhance their capability to capture long-range relationships^29^. While these advanced models improve segmentation accuracy, they also increase the number of model parameters, making them computationally expensive and requiring substantial annotated datasets for effective training^30^. The necessity of extensive annotated datasets is a significant drawback, especially in the medical domain where obtaining pixel-wise annotations is both costly and laborious^31^. The challenge of limited labeled data has driven the creation of training approaches, like semi-supervised learning, that can leverage data more effectively.

### Semi-supervised semantic segmentation approaches

Strategies like data augmentation, semi-supervised learning, and active learning are some of the existing ways to deal with the problem of minimal labeled data in segmenting the structures from histopathological images. Using a limited quantity of labeled data alongside a substantial collection of unlabeled data, semi-supervised learning techniques can enhance the efficiency of model training and can also be a cost-effective approach, making them a promising solution for addressing data scarcity in histopathological image segmentation^32^. This approach not only enhances the learning process but also helps in mitigating the impact of limited labeled data, enabling the wider implementation of neural network models in the examination of medical images^33^. In active learning we focus on identifying the most informative unlabeled data points for annotation, aiming to reduce the number of annotations required for good segmentation performance. However, it often necessitates annotating all objects in an image, which can be inefficient. Data augmentation^34^ artificially expands the training data by applying random transformations to existing labeled images, improving model generalization but not addressing the fundamental issue of limited labeled data. Semi-supervised learning tackles training challenges by incorporating both well-annotated data and vast amounts of unlabeled data. This enables the model to leverage a more comprehensive collection of data^35^. This is achieved by leveraging unlabeled data and automatically assigning pseudo-labels to it, effectively creating more training data^36^.

In a separate study^37^, the authors proposed BAWGNet uses boundary-aware units and wavelet-guided attention methods to improve nuclei segmentation in histopathology images. HER-CNN combines Mask R-CNNs with ResNet and Swin-Transformer, as well as edge awareness and synthetic data augmentation, to improve segmentation of irregularly shaped nuclei^38^. On histology datasets with few annotations, PG-FANet, a two-stage semi-supervised segmentation model with feature aggregation and inter- and intra-uncertainty regularization, provides state-of-the-art performance^39^. In an another study^40^, CKDNet is proposed which is a cascade knowledge diffusion network that combines coarse, classification, and fine segmentation with novel feature entanglement modules to improve skin lesion analysis. Extensive evaluations on ISIC datasets show superior performance without requiring ensembles or external data. DASC-Net combines domain adaptation and self-correction learning with unique attention and feature alignment algorithms to accomplish novel COVID-19 infection segmentation on CT images, overcoming domain shift difficulties^41^. A separate study offers a unique cell nucleus segmentation method, NST, leveraging Vision Transformers, as well as a new gastrointestinal cancer pathology dataset (GCNS), which achieves state-of-the-art performance with a Dice Score of 0.725^42^. The authors in^43^, proposed a Mean-Teacher-based hierarchical consistency enforcement (HCE) framework with a novel HC-loss to improve semi-supervised histological image segmentation, with results shown on the MoNuSeg and CRAG datasets.

Several recent advancements have been made in applying semi-supervised learning to histopathological image segmentation. Active learning combined with semi-supervised learning, such as the approach proposed in^44^, focuses on informative regions while utilizing unlabeled data, minimizing annotation needs and achieving high segmentation accuracy. Self-learning frameworks like Asynchronous Teacher-Student Optimization (ATSO)^45^ address issues like “lazy mimicking”. Consistency-based methods, such as the Dual Student architecture, use consistency constraints but can suffer from inaccurate predictions^46^. Unsupervised Domain Adaptation (UDA) frameworks like the Dual Teacher-Student (DTS) adapt models to different domains where labeled data is scarce^47^. Multi-task learning, cross-teacher training frameworks, and improved consistency learning with confidence-weighted loss have also shown promise. However, gaps remain, including the need for efficient annotation strategies, handling inaccurate pseudo-labels, and managing the complexity of multiple tasks and models.

## Methodology

### Overview

**Fig. 1:**
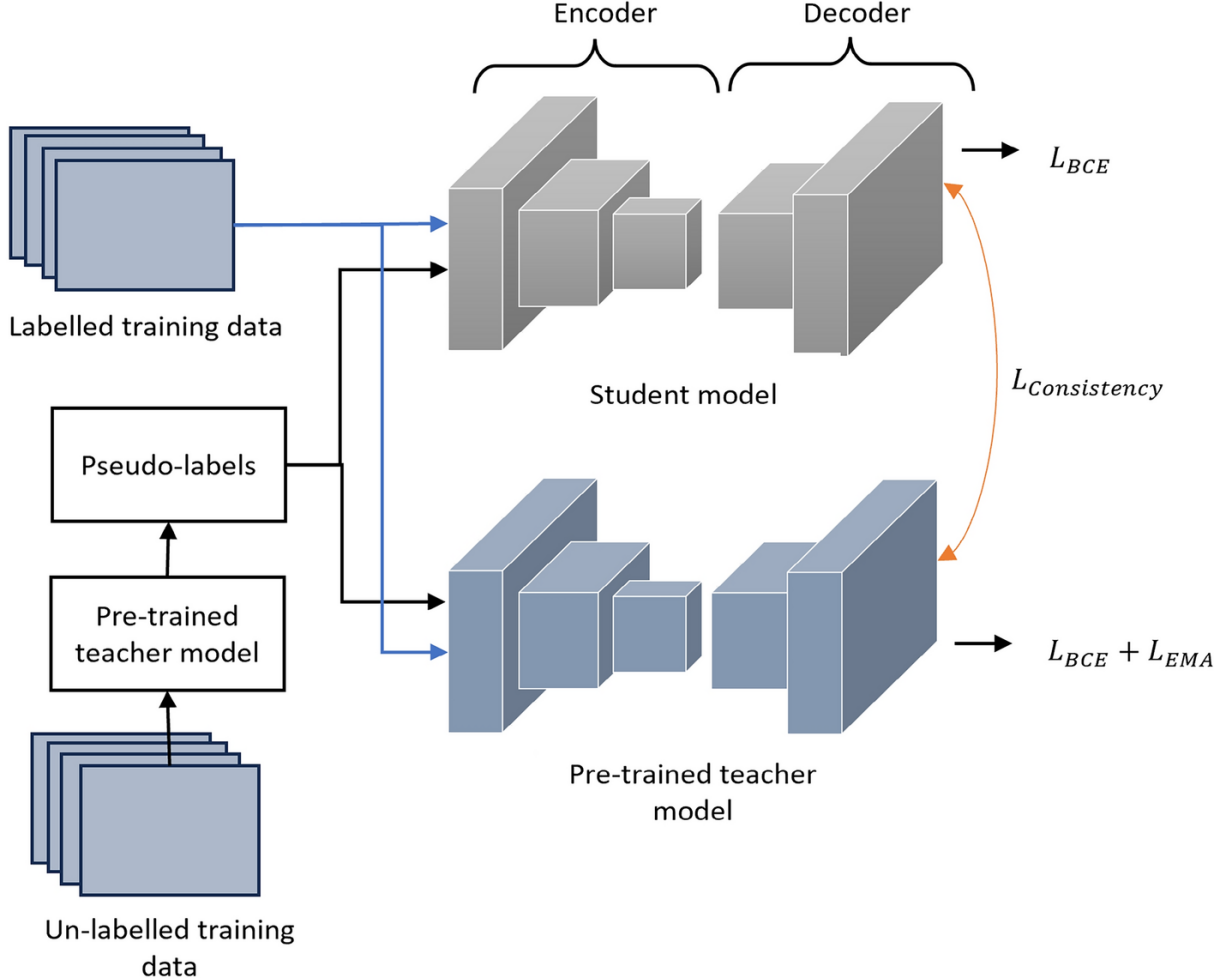
The overview of the proposed semi-supervised learning approach. The proposed framework uses a teacher model trained through self-supervised learning. Pseudo-labels are generated from this pre-trained model and used to train the student model.

We address the challenge of semi-supervised tissue semantic segmentation as a binary class segmentation task, with $C=\{c_1, c_2\}$. In semi-supervised learning, the training data is partially labeled, leading to a division of the training set into two subsets: $T=\{t_l,t_u\}$, where $t_l$ represents the labeled data and $t_u$ represents the unlabeled data. To effectively leverage the available training data, we employ a pseudo-label generation strategy utilizing the pre-trained teacher model. Initially, we train the teacher model ($\Phi$) on the labeled training data using a self-supervised learning approach. This framework tackles the challenge of limited labeled data in histopathological image segmentation by employing a two-model approach that leverages the full potential of available data. First, a pre-trained teacher model, trained using self-supervised learning, generates temporary labels (pseudo-labels) for the unlabeled data. These pseudo-labels essentially act as informed guesses about the category of each pixel in an unlabeled image. Next, a student model ($\omega$) is trained alongside the teacher model. This student model benefits from a richer learning environment by being exposed to both the original labeled data and the newly created pseudo-labeled data. This combined training significantly improves segmentation performance compared to using limited labeled data alone. To maintain alignment between the student model’s predictions and those of the teacher model, a consistency loss is incorporated. This loss function imposes a penalty on the student model when its predictions substantially differ from those of the teacher model, promoting agreement and mitigating potential errors introduced by the pseudo-labels. In essence, the teacher model acts as a guide for the student model, ultimately leading to improved segmentation accuracy. This consistency loss enhances the training process by encouraging the student model to closely follow the teacher model’s guidance, thereby improving overall model performance and robustness. In this work, hybrid functions, combining the advantages of other loss functions, are used because the effectiveness of utilizing a hybrid loss function has been shown in various works used in different networks in the literature^48–50^. The overview of the proposed framework for leveraging labeled and unlabeled data is shown in Fig. 1.

### Pre-processing: colour normalization

**Fig. 2:**
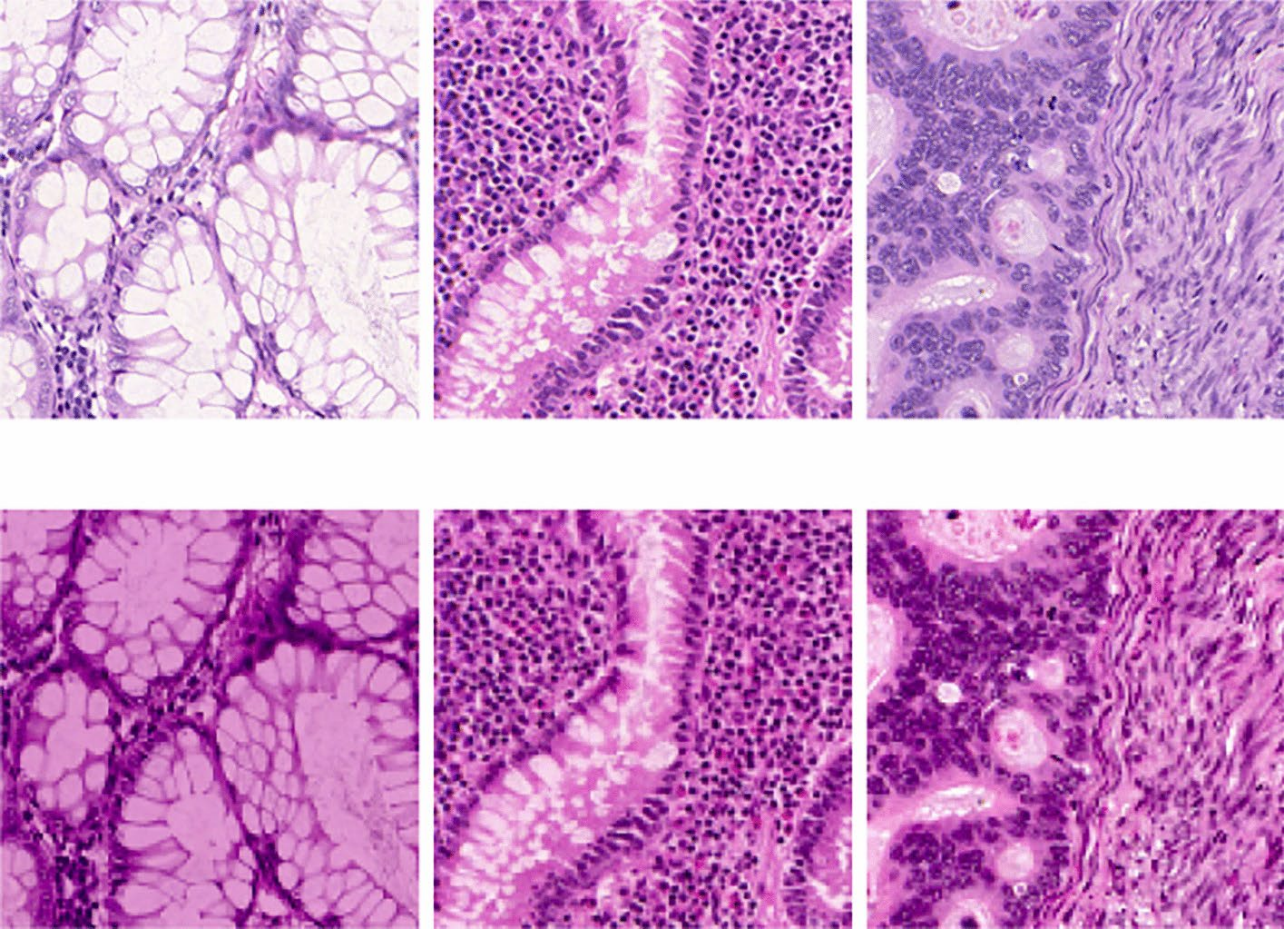
A few sample results of colour normalization using Reinhard method^51^.

The Reinhard method for color normalization is a widely used technique in the pre-processing of histopathological images^51^. This method addresses the variability in color that arises from differences in staining, lighting, and scanning conditions during image acquisition. The Reinhard technique transforms an image’s color distribution to closely match a reference image. This is achieved by converting both images to the Lab color space and then statistically aligning them. Specifically, the Reinhard method adjusts the target image’s L (lightness), a (green-red), and b (blue-yellow) channels to have the same average value (mean) and spread (standard deviation) as the corresponding channels in the reference image. This results in consistent and comparable color representations across different images, facilitating more accurate analysis and optimizing the performance of upcoming image processing tasks. In the present study, this method is adopted as a pre-processing step to reduce the variations in the colour distribution of these images. A few sample results of this step is shown in Fig. 2. The top row show few sample input images with varying colour distribution and the following row shows the results of colour normalization.

### Self-supervised training of teacher model

#### Design of teacher model

The design of the teacher model plays a crucial role in generating accurate pseudo-labels. Deep architectures typically require extensive training data to learn the model parameters effectively, while shallow architectures may lack powerful feature extraction capabilities. In this study, we meticulously designed an encoder-decoder based teacher model to balance these trade-offs. The designed architecture follows a U-Net-like design. Five convolutional blocks make up the encoder. Every block contains a convolutional layer and a subsequent batch normalization layer. The first block applies 32 filters of size $3 \times 3$, while the subsequent blocks apply 64, 128, 256, and 512 filters of the same size, respectively. Our proposed architecture employs a strategic design within each convolutional block to uncover significant patterns in the data. This design hinges on two key elements. Non-linearity is injected into the model through a ReLU activation function. This empowers the model to discover complex patterns in the data, going beyond the capabilities of a linear approach. Second, a max-pooling layer is strategically placed at the end of each block. This layer acts as a filter, identifying and retaining only the most prominent features for further analysis by subsequent layers. This interplay between non-linearity and feature selection empowers the network to effectively capture the essence of the data and progressively build more sophisticated representations as it delves deeper into the information processing layers. The decoder is designed with corresponding convolutional blocks. Each block includes an upsampling layer that doubles the feature map dimensions along the x and y axes, followed by a convolutional layer with $2 \times 2$ filters. An additional convolutional layer with $3 \times 3$ filters and ReLU activation function further refines the features. Batch normalization layers follow every convolutional layer to maintain stable learning. To mitigate information loss from max-pooling, and to ensure the decoder retains crucial details, we establish skip connections from the encoder. This allows the decoder to leverage high-resolution features from the encoder during reconstruction.

The final layer, a softmax layer, generates probabilities for each pixel representing its class membership. This architecture effectively balances depth and feature extraction capability, ensuring robust pseudo-label generation and enhancing the overall segmentation performance.

#### Self-supervised learning

A self-supervised training method is implemented to instruct the teacher model, facilitating its efficient learning from data that is only partially labeled. This training process spans multiple iterations to refine model performance progressively.

To equip the teacher model with knowledge, we first train it on the labeled dataset $t_l$ leveraging the provided labels. Subsequently, this well-trained teacher model is tasked with generating predictions for the unlabeled data $t_u$ . To promote continuous learning, we employ a progressive approach for the teacher model. In subsequent training rounds, we leverage the teacher model’s knowledge gained in the previous iteration to improve its capabilities. We achieve this by transferring the model’s weights (essentially its learned information) as a starting point (initialization) for retraining. This retraining happens on an enriched dataset that combines the original labeled data ($t_l$) with the unlabeled data ($t_u$) for which the pre-trained teacher model generated pseudo-labels (predictions treated as labels). This iterative approach allows the teacher model to progressively learn from both the well-defined labeled data and the additional information gleaned from the unlabeled data via pseudo-labels. We repeat this iterative process, wherein each cycle includes re-initializing the teacher model with updated weights, re-training on the entire dataset, and generating new pseudo-labels for $t_u$, until model performance convergence is achieved. This approach ensures that the teacher model continually improves its parameter learning and pseudo-label accuracy, thereby enhancing the overall quality of the segmentation task. This method is further described in Algorithm 1.

**Algorithm 1:**
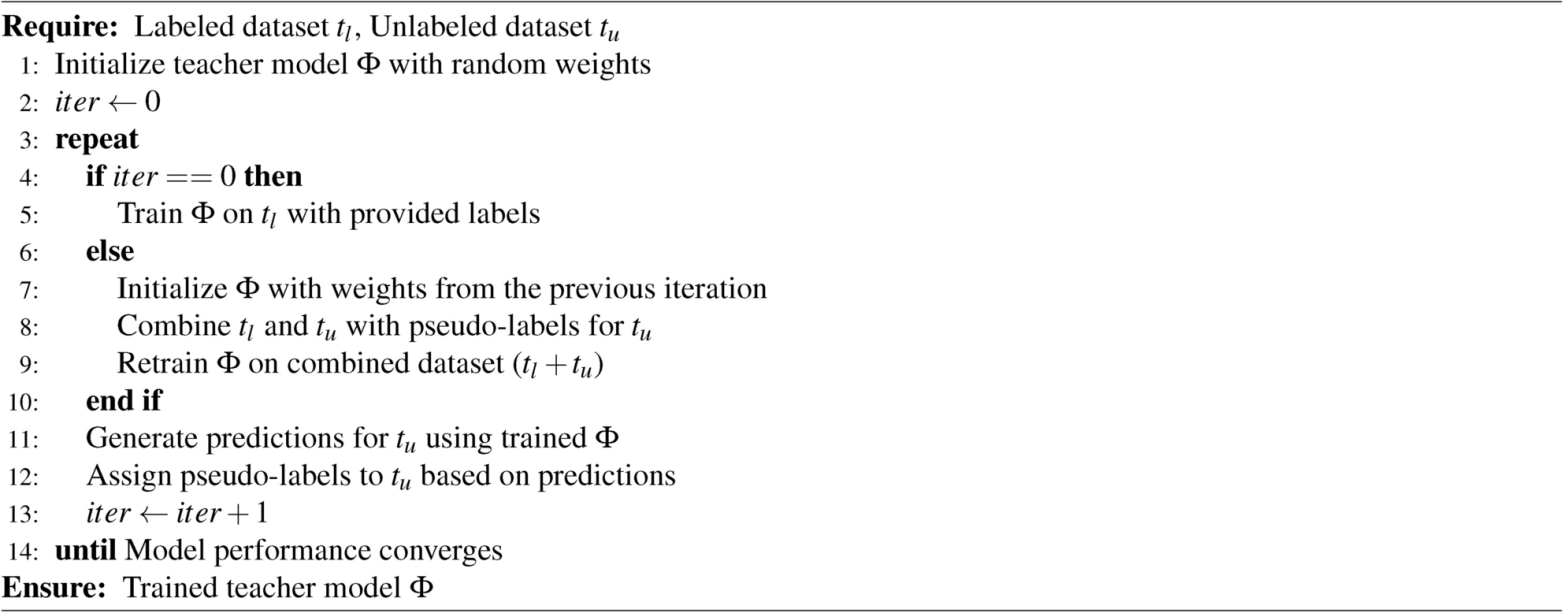
Self-supervised training strategy for teacher model.

### Generation of pseudo-labels

To produce final pseudo-labels for unlabeled training data, we employ a teacher model trained with a self-supervised learning technique. However, literature indicates that noisy pseudo-labels can lead to inaccurate training of the student model. To mitigate this issue, we adopt a thresholding strategy. For each pixel, we compare the teacher model’s predicted probability against a threshold value $\tau$. If the predicted probability for the foreground class exceeds $\tau$, we classify the pixel as belonging to the foreground. Otherwise, we classify it as a background pixel. This threshold value $\tau$ is determined experimentally to optimize the balance between precision and recall. This method aids in eliminating inaccurate labels, guaranteeing that only the teacher model’s high-confidence predictions are utilized in training the student model. This, in turn, enhances the precision and resilience of the student model, resulting in more dependable segmentation outcomes.

### Semi-supervised training of student model

#### Design of student model

In the present study, we designed the student model with a dual-encoder architecture, inspired by the approach proposed in^23^. The upper encoder consists of two convolutional blocks, each applying 64 and 256 filters with a kernel size of $5 \times 5$ to capture larger spatial information. Following each convolutional block, a max-pooling layer is applied which reduces the size of the feature maps by a factor of 4 in both width and height dimensions. Additionally, batch normalization follows each convolution layer to stabilize and accelerate the training process. The upper branch aims to capture extensive contextual information from the images. The lower encoder comprises four convolutional blocks, each containing two sets of convolutional layers. The number of filters in these blocks increases progressively from 64 to 512. Each convolutional block in this branch also includes a max-pooling layer that shrinks the feature map size by 2 in both width and height. At the bottleneck, we apply a convolutional layer with 1024 filters and a $3 \times 3$ kernel size. In a dual-encoder architecture, the feature maps generated by each encoder undergo concatenation. This combined feature map is subsequently fed into the decoder. The decoder is composed of four convolutional blocks, each incorporating an up-sampling layer to increase the feature map dimensions. Each block also includes two sets of convolutional layers with a $3 \times 3$ filter size. Similar to the U-Net architecture^20^, our proposed student model incorporates skip connections from the encoding layers to the corresponding decoding layers. However, we enhance these skip connections with spatial attention modules, which are detailed in section “Spatial attention module”, to capture relevant spatial features more effectively. The final layer is a softmax layer that produces a probability distribution of pixel classifications across different classes. This detailed architectural design ensures that the student model captures both fine-grained and large-scale features, enhancing its segmentation performance and robustness.

#### Spatial attention module

The spatial attention map is generated through a series of steps that enhance relevant spatial features in the encoding feature maps. Initially, we pass the encoding feature maps *F* through a $1 \times 1$ convolution layer, which averages the feature maps across channels:

1 $$\begin{aligned} F_{avg}=\zeta _{1 \times 1}(F) \end{aligned}$$

where, $\zeta$ represents the convolution operation. Next, we pass the output$F_{avg}$ through another $1 \times 1$ convolution layer with a sigmoid activation function, producing the spatial attention map *S*. The number of filters in this layer is set to 1:

2 $$\begin{aligned} S=\sigma (\zeta _{1 \times 1}(F)) \end{aligned}$$

Here, $\sigma$ denotes the sigmoid activation function. This spatial attention map *S* is then multiplied element-wise with the original encoding feature maps *F* to emphasize the relevant spatial features:

3 $$\begin{aligned} F_{att}=S\otimes F \end{aligned}$$

Finally, we concatenate the attention-enhanced feature maps $F_{att}$ with the decoding feature maps *D*:

4 $$\begin{aligned} D' = \Gamma (F_{att},D) \end{aligned}$$

where, $\Gamma$ represents the concatenation operation. This process ensures that the spatial attention mechanism highlights important regions in the feature maps, improving the segmentation performance by focusing on relevant spatial information.

#### Parameter optimization

The student model is trained along with the teacher model. The overall loss ($L_s$) for the student model is estimated as follows:

5 $$\begin{aligned} L_s=\mathscr {L}_{\text {BCE}}+\mathscr {L}_{\text {Consistency}} \end{aligned}$$

where, ${L}_{\text {BCE}}$ is the Binary Cross Entropy loss and $\mathscr {L}_{\text {Consistency}}$ represents the Consistency loss. In the present $L_{BCE}$ is given as follows:

6 $$\begin{aligned} \mathscr {L}_{\text {BCE}}(y, \hat{y}) = -\frac{1}{N} \sum _{i=1}^{N} \left[ y_i \log (\hat{y}_i) + (1 - y_i) \log (1 - \hat{y}_i) \right] \end{aligned}$$

where, $y_i$ is the true label for the $i$-th sample. The predicted likelihood for the $i$-th observation is expressed as $\hat{y}_i$. $N$ represents the number of samples. The consistency loss is used to ensure that the predictions of the model remain stable under different network perturbations. It can be defined as follows:

7 $$\begin{aligned} \mathscr {L}_{\text {Consistency}} = \frac{1}{N} \sum _{i=1}^{N} \left\| f(xi) - \tilde{f}(x_i) \right\| ^2 \end{aligned}$$

where, $f(x_i)$ is the prediction of the student model for the original input $x_i$. $\tilde{f}(x_i)$ is the prediction made by the teacher model for the original input $x_i$. $N$ represents the number of samples. $\left\| \cdot \right\| ^2$ denotes the squared Euclidean distance (or $\ell _2$ norm).

The teacher models weights are also updated while training the student model. To this end, we estimate the loss of teacher model as follows:

8 $$\begin{aligned} L_t=\mathscr {L}_{\text {BCE}}+\mathscr {L}_{\text {EMA}} \end{aligned}$$

where, $\mathscr {L}_{\text {BCE}}$ represents the BCE loss and $\mathscr {L}_{\text {EMA}}$ represent the exponential moving average loss. EMA smooths out the fluctuations in the model’s predictions by averaging them over time. This reduces the noise and variance in the training process, leading to more stable and reliable convergence. By incorporating past predictions, and by using EMA, the model becomes better at making accurate predictions on data it hasn’t encountered during training. In scenarios where the training data contains noisy labels, EMA helps in mitigating the impact of these noisy labels by smoothing the predictions over time. In the present study, following the approach proposed in^52^, we adopt EMA loss to update the weights of the teacher model and is given as follows:

9 $$\begin{aligned} \mathscr {L}_{\text {EMA}} = \frac{1}{N} \sum _{i=1}^{N} \left\| \hat{y}_i - \hat{y}_i^{\text {EMA}} \right\| ^2 \end{aligned}$$

where, $\hat{y}_i$ is the current prediction for the $i$-th sample. $\hat{y}_i^{\text {EMA}}$ is the EMA of the predictions for the $i$-th sample, updated as follows:

10 $$\begin{aligned} \hat{y}_i^{\text {EMA}} = \alpha \hat{y}_i + (1 - \alpha ) \hat{y}_i^{\text {EMA (previous)}} \end{aligned}$$

where, $\alpha$ is the smoothing factor $(0< \alpha < 1)$. $N$ is the number of samples. $\left\| \cdot \right\| ^2$ denotes the squared Euclidean distance (or $\ell _2$ norm).

## Results and discussion

### Performance metrics

We evaluated the proposed semi-supervised framework using a comprehensive range of assessment metrics. These include accuracy, precision, recall, F1-score, and mIoU. The most fundamental statistic is accuracy, which provides a direct measure of the model’s overall proportion of properly identified pixels. More specifically, precision focuses on how well the model reduces false positives. It determines the percentage of anticipated positive pixels that actually belong to the positive class. Unlike accuracy, recall measures the model’s ability to capture all important pixels. It measures the proportion of true positive pixels that were correctly categorized as positive. The F1-score strikes a balance between these two measurements, providing a more nuanced understanding of performance. The F1-score, calculated as the harmonic mean of recall and precision, is especially useful in situations where classes are imbalanced. Finally, mIoU considers the average overlap between the anticipated and actual positive regions in the image. High mIoU values suggest accurate segmentation masks, essential for testing and comparing models in histopathological image processing. This set of metrics offers a comprehensive evaluation of the framework’s image segmentation.

### Dataset

**Fig. 3:**
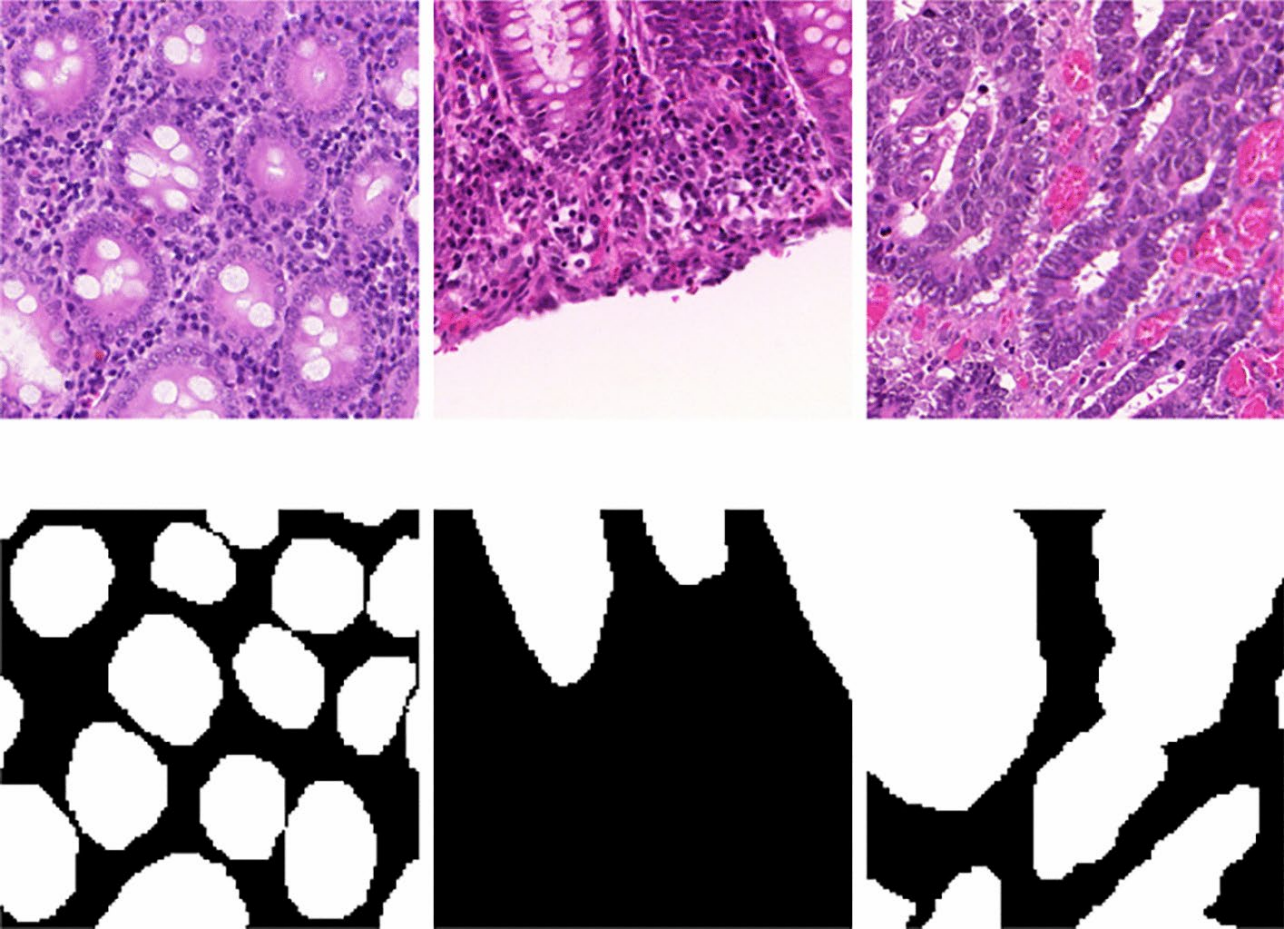
A few sample images, alongside their respective ground-truth labels from the dataset.

To assess the model’s performance, we used a publicly available dataset described in^53^. This dataset comprises 340 training images and 320 testing images, all resized to a uniform size of $128 \times 128 \times 3$ pixels. A sample of these images, alongside their respective ground-truth labels, is provided in Fig. 3 for illustrative purposes.

### Evaluation of teacher model

The choice of the teacher model is essential in creating pseudo-labels. To evaluate the performance of the teacher model, we conducted an experiment comparing it against popular architectures such as U-Net, ResUnet, DeepLabV3+, and PSPNet. All models were trained using the proposed self-supervised strategy to ensure consistency in evaluation. This experiment aims to identify the most effective model for generating accurate pseudo-labels, which are essential for improving the overall performance of the semi-supervised framework. The results of this comparative analysis are detailed in Table 1. From the table, it can be seen that ResUnet achieves an mIoU score of 0.43, which is close to that of the proposed model (0.45). However, the other models attained lower mIoU scores. As a result, their precision and recall values are also lower compared to those of the proposed model. Therefore, in scenarios with limited datasets, models like ResUnet, including the proposed model, are more suitable for generating pseudo-labels.

**Table 1:** Performance metrics comparison of teacher model with other contemporary methods. Significant values are in bold.

Iterations	Precision	Recall	F1-score	Accuracy	mIoU
Light U-Net	0.60	0.59	0.58	0.59	0.40
U-Net^20^	0.59	0.58	0.57	0.58	0.41
ResUnet^24^	0.61	0.61	0.61	0.61	0.43
DeepLabV3+^26^	0.45	0.44	0.44	0.44	0.37
PSPNet^54^	0.47	0.48	0.47	0.47	0.39
Proposed	**0.62**	**0.61**	**0.61**	**0.61**	**0.45**

### Evaluation of self-supervised training

**Fig. 4:**
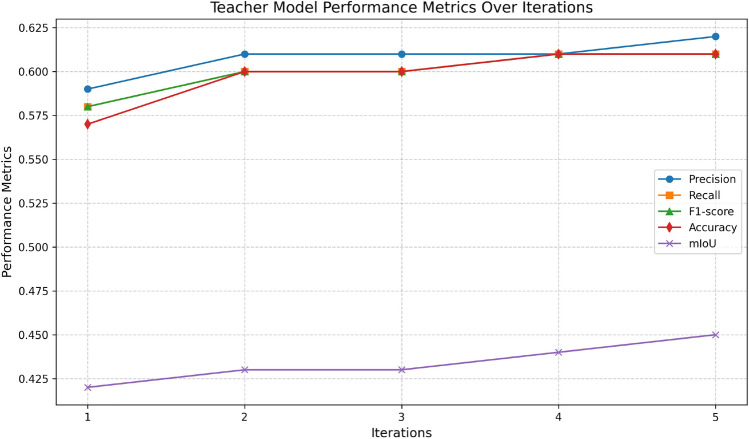
Evaluation of teacher model performance metrics across multiple training iterations.

**Fig. 5:**
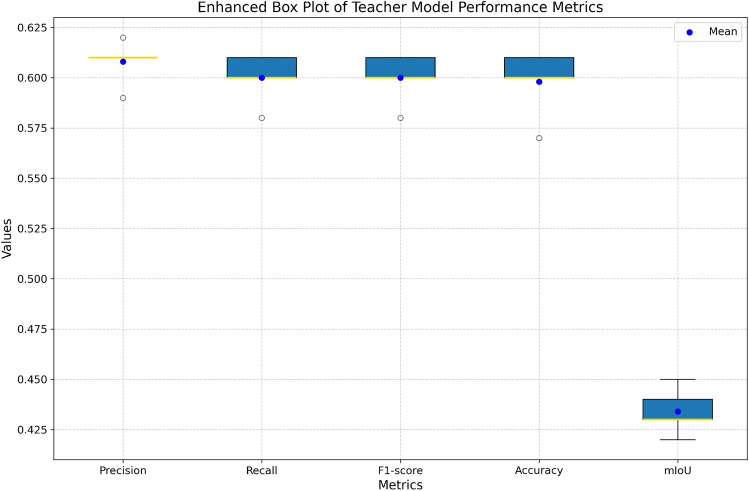
Box-plot of teacher model performance metrics across multiple training iterations.

This study tackles the challenge of training a teacher model with limited labeled data by employing a self-supervised learning approach. Self-supervised learning enables a model to extract meaningful patterns from unlabeled data, effectively generating additional training examples. This approach helps the teacher model become more robust and effective, even when working with a partially labeled dataset. In this regard, the number of iteration over which self-supervised learning is performed plays a prominent role in the training of the teacher model. Thus, an experiment was conducted to assess the performance of the teacher model at every iteration and 5 such iterations has been carried out. The Table 2 presents the performance metrics of a teacher model trained using a self-supervised learning strategy over five iterations. Figs. 4 and 5 compares graphically the metrics of teacher model over different iterations. The precision (yellow solid line) grows from 0.59 to 0.62 throughout the course of the iterations, indicating a steady improvement in the model’s capacity to forecast pertinent outcomes. The F1-score (pink dashed-dotted line) and recall (orange dashed line) both show an increasing trend, beginning at 0.58 and increasing to 0.61 by the fifth iteration. This shows that overall performance and sensitivity can be improved in a balanced way. The accuracy (purple dotted line) increases significantly from 0.57 to 0.61, which is consistent with the F1-score and suggests a general increase in the number of accurate predictions. mIoU (blue solid line), which represents improved overlap in the intersection over union metric, starts lower at 0.42 but gradually improves to peak at 0.45. Over the course of the iterations, all metrics demonstrate improvement, with the greatest values seen at iteration 5. While mIoU exhibits the slowest development but still shows progress, precision exhibits the most constant growth.

**Table 2:** Evaluation of teacher model performance metrics across multiple training iterations. Significant values are in bold.

Iterations	Precision	Recall	F1-score	Accuracy	mIoU
1	0.59	0.58	0.58	0.57	0.42
2	0.61	0.60	0.60	0.60	0.43
3	0.61	0.60	0.60	0.60	0.43
4	0.61	0.61	0.61	0.61	0.44
5	**0.62**	**0.61**	**0.61**	**0.61**	**0.45**

The distribution of each performance metric over the course of the five iterations is displayed in the box plot. The precision range is modest, with a small outlier at 0.59 and most values closely clustered around 0.61. Similar trends may be seen in recall, accuracy, and F1-score, with medians at 0.60 and small variances caused by outliers in the lower range. The range of mIoU values is greater, ranging from 0.42 to 0.45. In contrast to the other metrics, it exhibits greater fluctuation. With the exception of mIoU, which fluctuates significantly, this plot demonstrates that most metrics exhibit constant performance throughout iterations.

**Fig. 6:**
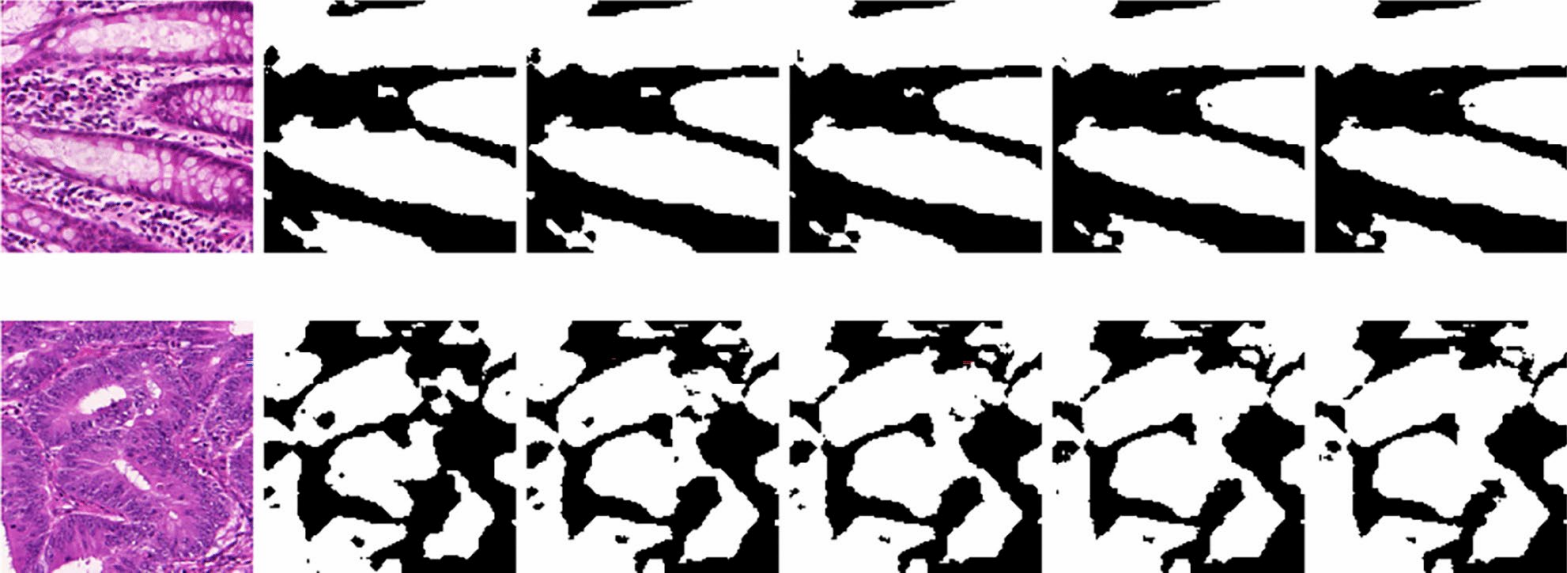
A few pseudo-labels generated by the teacher model over five different iterations.

We have also visualized the pseudo-labels generated by the proposed model for every iterations and has been shown in Fig. 6. It can be seen that, over the iterations, the quality of the pseudo-labels have improved. Further, the false positives generated by the proposed model is reduced which are highlighted in the figure. This further validates the benefits of self-supervised learning for training the teacher model.

### Evaluation of pseudo-labels

Evaluating the quality of pseudo-labels produced by the teacher model is crucial to ensure that they are reliable and useful for training the student model. In the present study, we have set $\tau =0.7$. An experiment is carried out for evaluating the reliability of pseudo-labels produced by the teacher model. To this end, we have visualized the pseudo-labels, as shown in Fig. 6. Furthermore, we have also performed uncertainty estimation using the Monte Carlo (MC) Dropout technique. The approach includes conducting numerous forward passes through the network while enabling dropout during inference. Initially, we train the teacher model incorporating dropout layers. Unlike standard practice where dropout is disabled during inference, for MC Dropout, dropout is kept enabled during inference. This is crucial for generating diverse predictions for the same input. Pass the input data through the network multiple times (e.g., 30 or more forward passes), each time with dropout enabled. This will result in a set of different predictions due to the stochastic nature of dropout. By analyzing the model’s predictions, we can gain valuable insights into its performance.

**Fig. 7:**
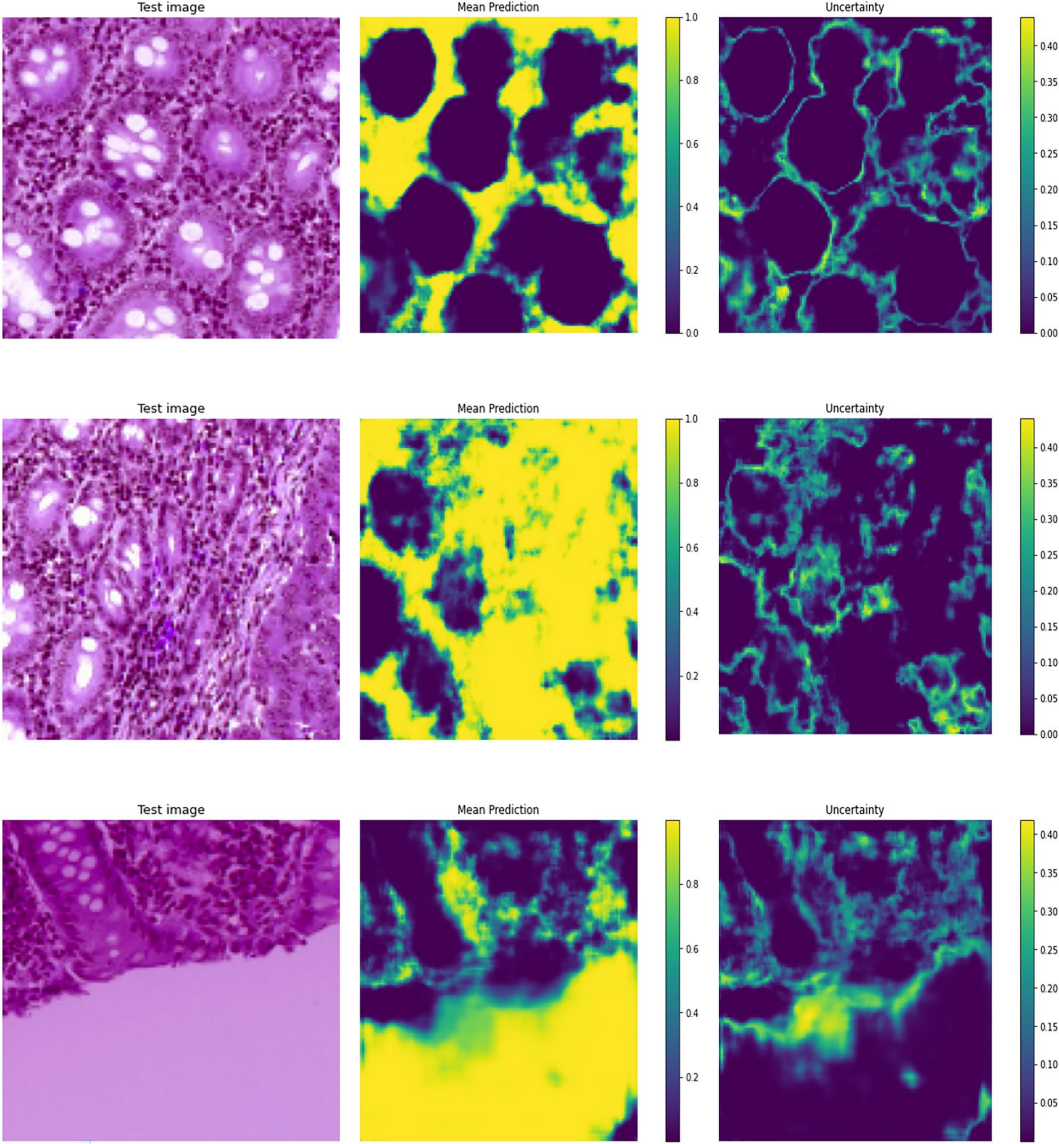
A few sample results of uncertainty estimation using Monte Carlo dropout method.

The Fig. 7 displays two types of heatmaps after three rows, each of which represents a distinct test image in the first column: The model’s average predictions for every pixel in the test images are displayed in these heatmaps. The heatmaps’ hues range from yellow (high prediction) to dark purple (poor prediction). Darker (purple) regions denote lesser confidence in the projected label, whereas regions with high values (closer to yellow) suggest places where the model is more sure about its predictions. The different tones of purple and yellow indicate that the model is more certain about some locations while being unsure about others. As can be seen from the second column, the model is very certain that the pixels in bright yellow regions are background-related (e.g., non-relevant tissue or empty spaces in the input images). Dark purple areas show a low degree of confidence in the background label assignment, indicating that the model either considers these areas to be in the forefront or is uncertain. The areas of darker purple are probably those where the model has trouble differentiating background tissue structure from actual tissue structure. All test images have clear, distinct background regions that the model confidently labels. The difference in color intensity (purple vs. yellow) suggests that the model is less confident and struggles more in areas with intricate textures or background-to-tissue transitions. Similarly, the third column shows that numerous regions within the cellular structures (foreground) have a mix of yellow and purple, indicating varying levels of confidence. There is strong confidence in those particular foreground locations, as indicated by the more distinct tissue sections (purple). There are more scattered yellow areas in the second row, which suggests that the model is less certain about how to distinguish foreground features in this image. This is probably because the structures are unclear or have complicated textures. When marking tissue structures in the foreground as opposed to the background, the model frequently shows greater ambiguity, especially in regions with complex textures or ill-defined boundaries.

### Evaluation of semi-supervised learning

**Fig. 8:**
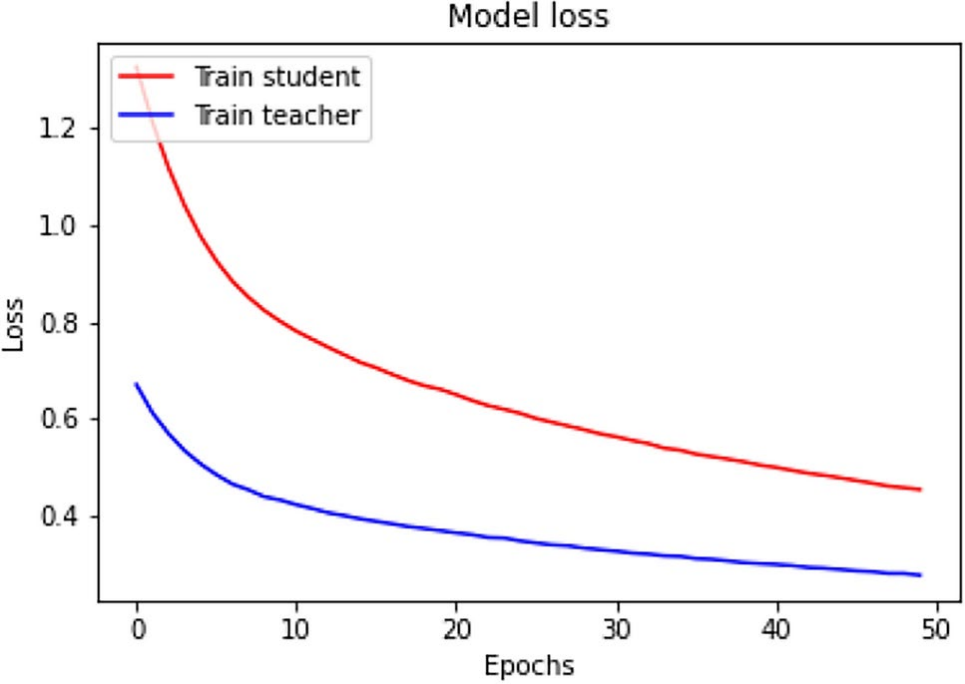
The training loss plots of the teacher and student models.

To assess our proposed semi-supervised learning framework’s effectiveness, we benchmark it against established approaches like self-supervised learning, pseudo-label generation, and consistency learning. Additionally, we design multiple variations of our framework for an extensive ablation study. In our evaluation, the self-supervised learning strategy employs the proposed student model and follows the methodology detailed in section “Methodology”. For the pseudo-label generation method, we utilize the proposed teacher model to create pseudo-labels. The student model is subsequently trained on the training dataset using both the actual labels and the pseudo-labels, optimizing the Binary Cross Entropy loss function. For the consistency regularization method, we modify the student model by incorporating an auxiliary decoder and train it using a consistency loss. We also develop two variants of the proposed semi-supervised framework. The first variant involves training the student model on the dataset without employing the teacher model or consistency loss. The second variant includes a teacher model where the weights are updated using the Dice loss. We present various performance metrics for these methods in Table 3, providing a comprehensive comparison of our framework with existing approaches. The training loss plots of the teacher and student models are shown in Fig. 8. The Table 3 compares the performance metrics of different semi-supervised semantic segmentation approaches for tissue segmentation from histopathological images. The metrics include precision, recall, F1-score, accuracy, and mean Intersection over Union (mIoU). This method shows relatively balanced performance across all metrics, with precision and recall both around 0.70–0.71. However, its mIoU is the lowest among the methods, indicating less effective segmentation in terms of overlap between predicted and actual segments. Pseudo-label generation method demonstrates an improvement over self-supervised learning, especially in precision and accuracy, suggesting better prediction quality and overall performance. The mIoU also indicates better segmentation performance compared to the self-supervised baseline. Consistency learning performs slightly better than self-supervised learning but falls short compared to pseudo-label generation. It shows a balanced performance but with moderate improvements in mIoU and F1-score. Variant-1, which trains the student model without the teacher model or consistency loss, performs comparably to pseudo-label generation. It achieves the highest recall (0.75) among the methods, indicating its effectiveness in identifying true positives. The proposed approach outperforms all other methods across all metrics. It shows the highest precision,recall, F1-score, accuracy, and mIoU, indicating superior segmentation performance and better overall effectiveness in identifying and segmenting tissue structures in histopathological images.

**Table 3:** Performance metrics comparison of various training strategies. Significant values are in bold.

Methods	Precision	Recall	F1-score	Accuracy	mIoU
Self-supervised learning^55^	0.70	0.71	0.71	0.71	0.59
Pseudo-label generation^36^	0.74	0.72	0.73	0.74	0.62
Consistency learning^56^	0.72	0.73	0.72	0.72	0.61
Variant-1	0.74	0.75	0.75	0.74	0.62
Variant-2	0.74	0.72	0.72	0.72	0.61
Proposed approach	**0.79**	**0.78**	**0.78**	**0.78**	**0.64**

**Fig. 9:**
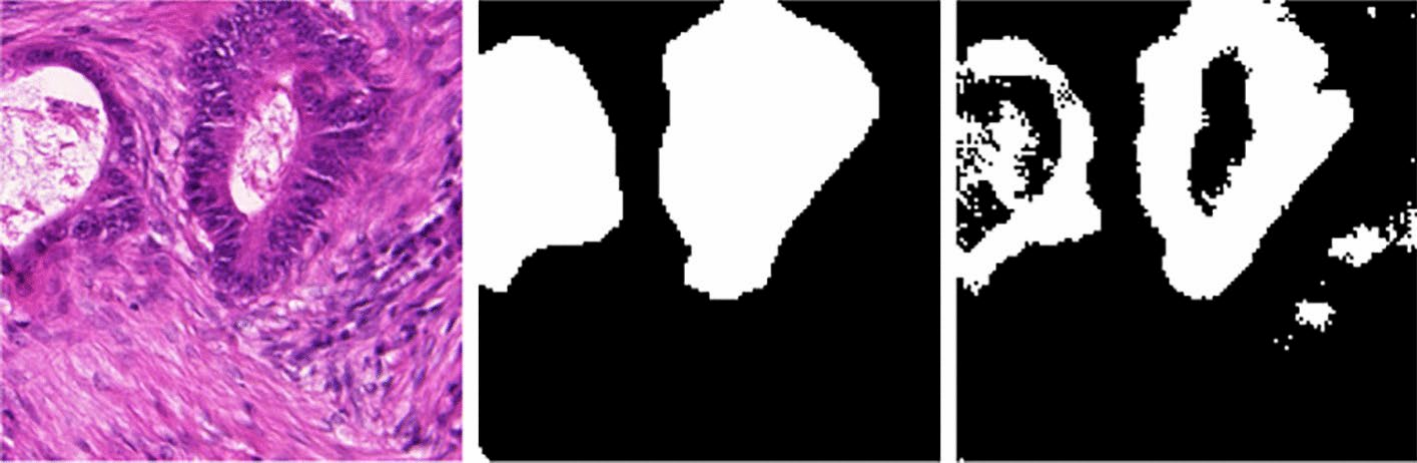
The input and its corresponding ground-truth image are shown in the first two columns. The last column shows the output of the proposed approach. As seen from the figure, the model fails to segment the tissue structure accurately.

In some instances, the proposed model does not achieve precise segmentation of the tissue regions, as demonstrated in Fig. 9. The figure highlights the model’s difficulty in accurately delineating the region of interest, primarily due to the heterogeneous structure of the tissue samples. This limitation arises from the semi-supervised approach employed by the model, which relies on pseudo-labels and consistency regularization for learning. While pseudo-labels provide a means to utilize unlabeled data, their inherent noise and inaccuracies can propagate through the training process, leading to suboptimal segmentation results. Additionally, the consistency regularization, though effective in enforcing stable predictions across augmented views, may struggle to generalize well to regions with highly variable or complex tissue patterns. These challenges could be mitigated by adopting a deeper architecture capable of extracting more comprehensive and discriminative features, thereby enhancing the model’s ability to handle structural heterogeneity in tissue samples.

## Conclusion

This research presents a novel semi-supervised learning framework for histopathological image segmentation, leveraging both labeled and unlabeled data to improve segmentation accuracy. By introducing a lightweight CNN to create pseudo-labels as a teacher model and utilizing a Monte Carlo dropout technique for uncertainty estimation, we ensure robust pseudo-label generation. The proposed student model is designed to capture both spatial and contextual features effectively, enhancing the precision of segmentation maps. Our innovative semi-supervised framework, which employs consistency regularization, has demonstrated promising results on a public dataset. This approach significantly reduces the reliance on large, fully annotated datasets, addressing a critical challenge in the medical imaging domain where acquiring pixel-wise annotations is both costly and laborious.

Comparative evaluations against popular semi-supervised approaches, such as self-supervised learning, pseudo-label generation, and consistency learning, as well as various ablation studies, highlight the superior performance of our proposed model. The results indicate substantial improvements across all evaluation metrics, validating the effectiveness of our methodology. Our research contributes to the advancement of histopathological image segmentation by providing a more efficient and accurate framework. This framework holds potential for broader applications in medical image analysis, ultimately contributing to enhanced diagnostic and research capabilities. However, the proposed semi-supervised model faces limitations in handling regions with high structural heterogeneity due to the inherent noise in pseudo-labels, which can affect segmentation accuracy. Additionally, the reliance on consistency regularization may limit the model’s ability to generalize effectively in scenarios involving highly complex or variable patterns. As a future work, an effective denoising method, such as in^57,58^, can be integrated into the proposed approach because images are mostly noisy and an effective denoising can significantly eliminate the negative impact of noise. A potential future work can be an evaluation of the proposed approach for segmentation of organs from MRIs, particularly segmentation of kidneys and the liver from abdominal MRIs because the similarity in adjacent tissues makes their segmentation challenging. Although there are various deterministic or atlas-based methods^59–64^, an effective CNN-based method is needed.

## Data Availability

The datasets analysed during the current study are available in the github repository, https://github.com/lyndonchan/hsn_v1.
